# A Novel Disposable Bamboo Biochar-Based Electrochemical Sensor for Detecting the Nonsteroidal Anti-Inflammatory Drug Flufenamic Acid in Environmental Samples

**DOI:** 10.1021/acsomega.5c06160

**Published:** 2025-08-05

**Authors:** Francisco Walison Lima Silva, Luís Eduardo da Conceição Teixeira, Cassiano Augusto Rolim Bernardino, Claudio Fernando Mahler, Renata Coura Borges, Ricardo Erthal Santelli, Fernando Henrique Cincotto

**Affiliations:** † Departamento de Química Analítica, Instituto de Química, 28125 Universidade Federal do Rio de Janeiro, Rio de Janeiro 21941-909, Brazil; ‡ Departamento de Engenharia Civil, COPPE, Universidade Federal do Rio de Janeiro, Rio de Janeiro 21941-914, Brazil; § Departamento de Solos, Instituto de Agronomia, 67825 Universidade Federal Rural do Rio de Janeiro, Rio de Janeiro 23897-000, Brazil; ∥ National Institute of Science & Technology of Bioanalytics (INCTBio), Campinas, São Paulo 13083-970, Brazil

## Abstract

This study presents an electrochemical platform for the detection of flufenamic acid using a bamboo biochar-modified screen-printed electrode (denoted as SPE/BCB). The proposed sensor exhibited high analytical performance, with a sensitivity of 2.30 μA/μmol L^–1^ and an ultralow detection limit of 1.3 nmol L^–1^, across a broad linear range (0.05–13.32 μmol L^–1^). Compared with conventional electrodes such as glassy carbon, carbon paste, and modified pyrolytic graphite electrodes, the SPE/BCB sensor offers advantages in terms of cost-effectiveness, ease of fabrication, and disposability. The incorporation of bamboo biochar enhances the electrochemical performance while providing an environmentally friendly approach. Furthermore, the sensor demonstrates excellent selectivity, remaining unaffected by common organic interferents, making it suitable for environmental applications. Its ability to accurately quantify FFA in complex aqueous matrices, including river and tap water, highlights its potential as an effective tool for environmental monitoring.

## Graphical abstract

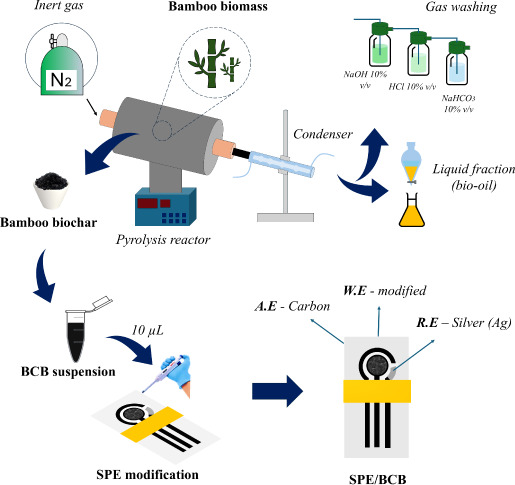

## Introduction

Nonsteroidal anti-inflammatory drugs (NSAIDs) are among the most widely used medications worldwide, owing to their beneficial effects in both short- and long-term therapeutic treatments due to their analgesic and antipyretic properties.^1^ In this context, flufenamic acid (FFA), chemically known as *N*-(α, α, α-trifluoro-m-tolyl) anthranilic acid, is recognized for its anti-inflammatory, analgesic, and antipyretic effects. It is commonly applied in the management of inflammation, pain relief, antirheumatic therapy, and peri-articular and soft tissue disorders.^2^ The chemical structure of FFA and its possible radical are presented in Scheme 1.

**Scheme 1:**
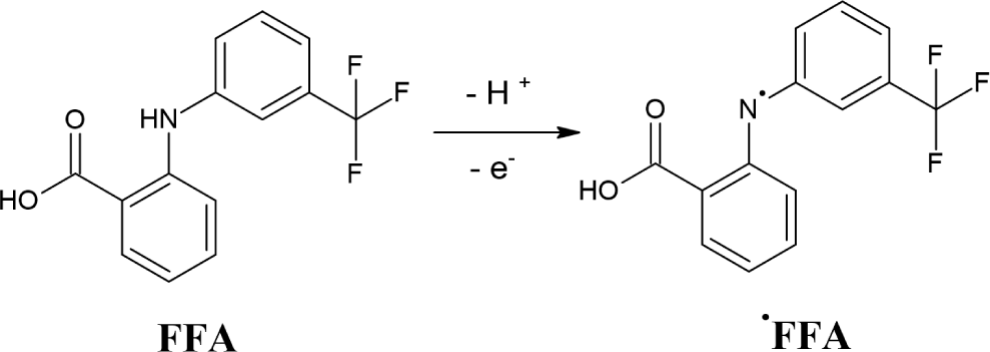
Mechanism of the Electrooxidation of Flufenamic Acid (FFA) to a Radical

Due to its fluorinated structure, FFA is nonbiodegradable and represents a significant environmental pollutant, particularly in aquatic ecosystems. As a member of the fenamate class, FFA undergoes metabolism primarily via hydroxylation and glucuronidation, and around 50% of the administered dose of FFA is excreted in human urine, as its half-life is approximately 3 h.^3^ A considerable portion of the drug is excreted unchanged, contributing to its continuous release into wastewater systems. Conventional wastewater treatment plants are often ineffective in fully removing pharmaceutical residues, allowing FFA to persist and enter natural water bodies. According to Canona et al.,^4^ FFA was detected in tap water and river water at average concentrations of 16 and 21 ng/l, respectively.

Environmental contamination through the excretion of NSAIDs in urine has become a growing concern. These pharmaceutical residues contaminate water bodies and river water (surface water), affecting aquatic ecosystems and human health due to exposure to and consumption of contaminated water.^5^ In addition, it has been reported in the literature that FFA presents toxicity to aquatic plants such as *Chlamydomonas reinhardtii* (green algae) by inhibiting photosynthesis.^6^ According to Nadanaciva et al.,^7^ FFA affects multiple phenotypic end points associated with hepatic and gastrointestinal toxicity in *Danio rerio* (zebrafish).

Various methods have been used for the determination of FFA and its derivatives, such as chromatography,^8,9^ spectrophotometry,^10–12^ and capillary electrophoresis,^13,14^ for example. These methods, although common, have limitations, such as high-cost analysis, complex sample preparation, rigorous protocols, and time-consuming analysis. Electrochemical sensors have been widely used due to their high selectivity and low cost. The literature reports various electrode modifications, such as the use of nanomaterials, conducting polymers, biomolecules, and metal oxides, to enhance analytical performance. These modifications enable efficient detection of different analytes in complex matrices.^15–17^ Electrochemical methods offer several advantages for the detection of FFA, including simplicity, rapid analysis, and high sensitivity. Various materials have been reported in the literature for sensor surface modification, such as poly-*N*-acetylaniline^18^, multiwalled carbon nanotubes,^19^ oxide nanoparticles,^20–22^ and dodecyltrimethylammonium chloride.^23^ Examples of electrodes used for these sensors include glass carbon electrodes, pyrolytic graphite electrodes, and carbon paste electrodes. In addition, the use of the biochar obtained by pyrolysis from waste woody biomass has been presented as a green alternative material for the electrochemical and adsorption detection of FFA, both in the presence and absence of cetyltrimethylammonium bromide.^24^

Electroanalytical detection of FFA has gained attention due to its sensitivity and specificity, typically utilizing conventional electrodes that are modified to enhance performance. In this context, the use of screen-printed electrodes (SPEs) has emerged as a promising alternative. SPEs offer advantages such as low cost, easy fabrication, and disposability, making the process more accessible and efficient for large-scale analyses and low sample volumes per analysis.^25^ Furthermore, the modification of these electrodes with various materials, particularly biochar, can provide enhanced sensitivity and selectivity for FFA, especially in environmental samples. Overall, the versatility of SPEs modified with carbon-based materials derived from pyrolyzed biomass makes them valuable tools for FFA detection, offering improved sensitivity and practicality. Biochar, a carbon-rich material, is a substance made through the pyrolysis of biomass and is used in pollution remediation and wastewater treatment.^26^ According to Liu et al.,^27^ the slow pyrolysis process operates at temperatures from 300 to 800 °C, and the main product obtained is biochar (yield 35–50%). It has a broad electroanalytical applicability due to its potential to improve the detection of pollutants, conductivity, large surface area, and analytical stability.^28^

In this context, the present study presents the development of a low-cost screen-printed electrode prepared from conductive carbon and silver ink, adhesive, and transparency sheet, modified with bamboo biochar (BCB) obtained by slow pyrolysis, denoted as SPE/BCB, for determination of the NSAID flufenamic acid (FFA) in environmental samples. The study highlights the use of low volumes per analysis (50 μL), as well as the disposability and sensitivity of the proposed sensor, in addition to other characteristics of electrochemical sensors.

## Experimental Section

### Reagents and Apparatus

The analytical-grade reagents used in the studies included flufenamic acid (FFA), hydrochloric acid, potassium hexacyanoferrate (II) and (III), sodium hydroxide, monobasic sodium phosphate, dibasic sodium phosphate, potassium chloride, and ethanol PA, all sourced from Sigma-Aldrich (Germany). Ultrapure water with a resistivity greater than 18.2 MΩ·cm was obtained from the Merck Milli-Q Reference system. The screen-printed electrodes (SPE) were fabricated using carbon ink (C2030519P4) in the working electrode (3.0 mm diameter) and the counter electrode. The reference electrodes were painted with Ag/AgCl ink—C2130905D3. These inks were obtained from Gwent Electronic Materials Ltd. (United Kingdom). The PGSTAT 204 potentiostat/galvanostat was used for electroanalytical experiments. The Quantachrome Modelo NOVA 2200 and the FEI Magellan 400 microscope were used for the morphological characterization of the bamboo biochar.

### SPE/BCB Preparation

The screen-printed electrodes (SPEs) were fabricated through the following steps: a vinyl adhesive sheet was applied to a transparency sheet (A4 size), which was then processed in a cutting printer. The vinyl sheet was removed to create the mask, followed by coating it with carbon ink. The mask was then heated at 60 °C for 30 min to ensure the ink dried properly. Afterward, silver ink was applied to the designated area for the reference electrode, and it was dried in an oven at 56 °C for an additional 20 min. The excess vinyl mask was removed, and the screen-printed electrodes were carefully cut out. A vinyl tape was placed over the electrode contacts either before or after the modification process, depending on the analysis needs.

The bamboo biochar was produced by slow pyrolysis method, occurred at 600 °C in a nitrogen gas atmosphere, with a heating rate of 15 °C min^–1^ and a residence time of 2 h. The experiments were carried out using a bench-scale reactor equipped with a temperature control system and a borosilicate tube. The gaseous products generated at 600 °C were condensed within a temperature range of 5–15 °C. The uncondensed gases were captured in washing bottles containing sodium hydroxide (10% w/v), hydrochloric acid (10% v/v), and sodium bicarbonate (10% v/v) solutions. The condensed products were separated into pyrolysis water and bio-oil using a separation funnel.^29^ Once cooled, the remaining biochar (denoted as BCB) was collected and characterized to assess its potential for electrooxidation of the FFA drug.

To modify the SPEs, a suspension containing 1 mg of BCB in 2 mL of water was prepared; 10 μL of this suspension was applied to the working electrode surface. The modified electrodes were then allowed to dry at room temperature. The process of biochar bamboo fabrication and modification of SPEs is shown in Scheme 2.

**Scheme 2:**
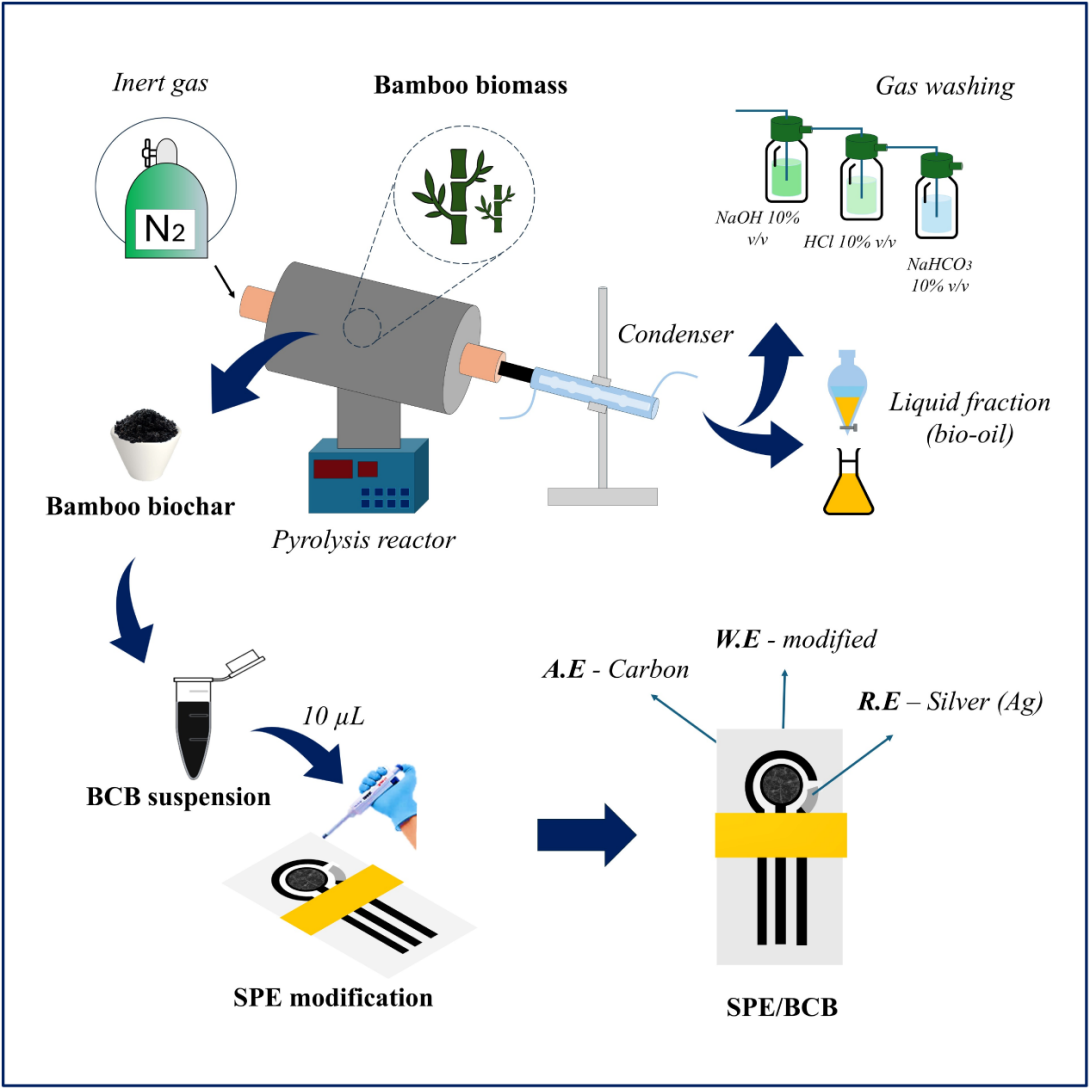
Pictorial View of the Bamboo Biochar (BCB) Production and the Screen-Printed Electrode (SPE) Modification

### Preparation of Real Samples

The SPE/BCB sensor was used to determine the FFA drug in environmental samples. The FFA standard solution was added to tap water samples B, C, and D, whose final concentrations were 0.4, 5.68, and 8.52 μmol L^– 1^, respectively. Tap water sample A was prepared without the addition of FFA. The river water used in the analysis was collected in Porto Firme, Minas Gerais, Brazil (20°39′44″ S, 43°5′1″ W). Four river samples were prepared for analysis. FFA standard was added to samples F, G, and H (final concentrations were 0.4, 5.68, and 8.52 μmol L^– 1^, respectively). Sample E was obtained without the addition of FFA. All real samples were prepared in 0.1 mol L^–1^ PBS buffer (pH 7).

## Results and Discussion

### Material Characterization

The biochar derived from bamboo after pyrolysis treatment was analyzed using the Brunauer–Emmett–Teller (BET) method, yielding a specific surface area of 125.7 m^2^/g, and a total pore volume of 0.09 cm^3^/g. The average pore diameter of 26.5 Å was obtained by the Barrett, Joyner, and Halenda (BJH) method. Additionally, morphological characterization using scanning electron microscopy (SEM) revealed the characteristic porous structure typical of biochar (Figure 1A,B). The SEM analysis, complemented by energy-dispersive X-ray spectroscopy (EDS) at four different points, identified the presence of carbon (C), oxygen (O), and silicon (Si) in the selected regions. In Figure 1B, at points 2, 3, and 4, C and O were the predominant elements. In point 1, silicon (Si) is linked to the formation of silica (SiO_2_) following thermal treatment. Additionally, silica-based structures observed within and on the pore surface of the bamboo biochar may be related to phytoliths-Si, which are rigid, microscopic silica deposits that form within plant tissues and persist after decomposition, contributing to the mineral content of the resulting biochar.

**Figure 1:**
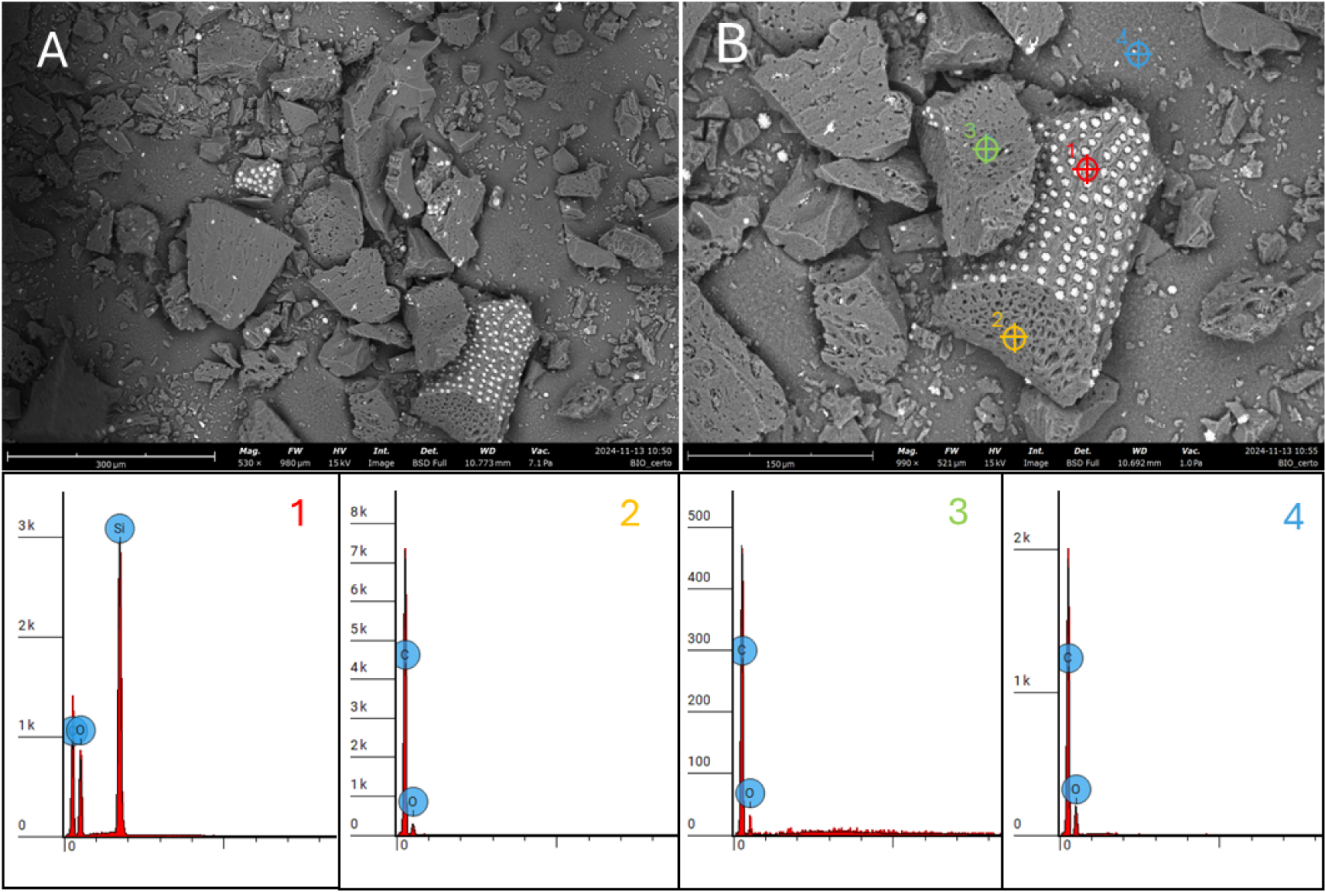
(A) and (B) SEM images of the bamboo biochar and the corresponding EDS spectra for the four points (1-red, 2-orange, 3-green, and 4-blue) indicated in the SEM image (B).

The infrared (IR) spectrum of biochar produced through the pyrolysis of bamboo at 600 °C reveals valuable information about its molecular composition. The analysis of the absorption peaks indicates the presence of various functional groups and structural characteristics. The peak at 3338 cm^–1^ suggests the presence of OH and NH groups, indicative of hydroxyls and amines, respectively. C–H stretching is also observed in the region around 2920 cm^–1^. The absorption at 1580 cm^–1^ suggests the presence of C=C stretching associated with aromatic rings, as well as C=O stretching and N–H bending, characteristic of amides. The absorption at 1374 cm^– 1^ may indicate an interaction between OH bending and C–O stretching, possibly derived from fatty acids or other oxygenated organic compounds. Vibrations at 880 cm^–1^ may be attributed to aromatic C–H deformations, and the peak at 756 cm^–1^ may be associated with aliphatic C–H deformation or monosubstituted C–H bending.^29,30^ In general, the presence of functional groups such as OH and NH suggests potential redox interactions at the solid–liquid interface, influencing charge transfer processes. The C=C double bonds indicate the propensity of biochar to participate in redox reactions, thereby affecting the sensor’s ability to detect specific species. Methylene and oxygenated groups impact the surface’s hydrophobicity and selectivity, while C–H bond deformations in rings and carbon chains modulate electrical conductivity and reactivity at the electrochemical interface. The results are shown in Figure 2.

**Figure 2:**
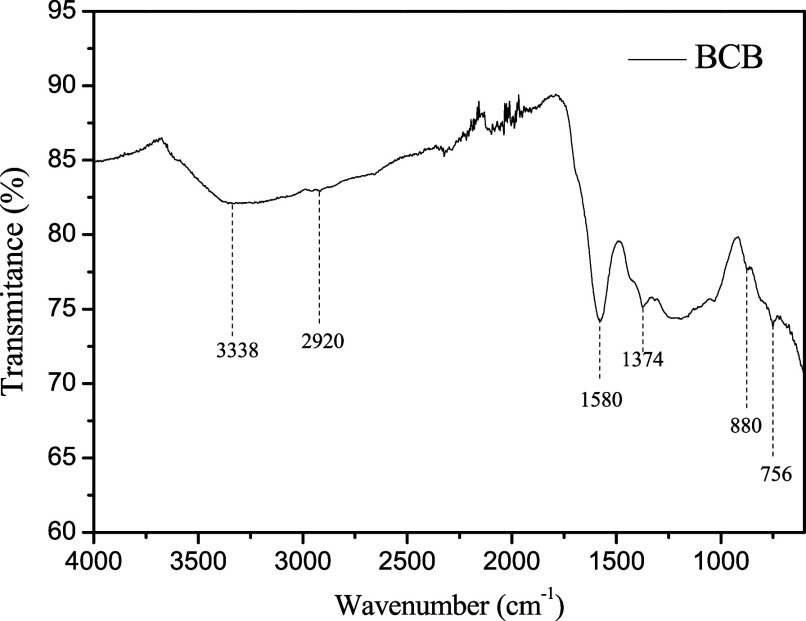
FTIR spectrum of BCB material.

### Electrochemical Measurements

Bare SPE and SPE/BCB were used to evaluate the electrochemical behavior of FFA by using differential pulse voltammetry (DPV) and cyclic voltammetry (CV) techniques. Initially, CV analyses were performed with a neutral aqueous solution of 50.0 μmol L^–1^ FFA in 0.1 mol L^–1^ PBS. According to the voltammograms shown in Figure 3A, the SPE/BCB demonstrated a greater contribution to the oxidoreduction of FFA compared to the bare SPE, exhibiting peak currents of 7.0 μA and 12 μA, corresponding to peak potentials of 0.45 and 0.42 V vs ref (Ag/AgCl in KCl_sat_), respectively. Additionally, as shown in Figure 3B, FFA displayed peak I, corresponding to the irreversible electrooxidation at 0.42 V, followed by an electroreduction peak IIa at 0.115 V, representing the cathodic response of a product from the first oxidation. Peak–peak separation (Δ*E*_p_) between peak I and IIa is >200 mV, which indicates irreversible behavior. Peak IIa is dependent on peak I (peak potential of 0.174 V). Finally, peak IIb represents the anodic response dependent on the reduction (peak IIa), as noted by its absence in cycle I, indicating a reversible process due to Δ*E*_p_ of 59 mV. These results are also presented in the literature for carbon-based electrodes.^23^ Figure S1 shows the absence of additional peaks when comparing the cycles from −0.2 to 1.0 V and from −1.0 to 1.0 V.

**Figure 3:**
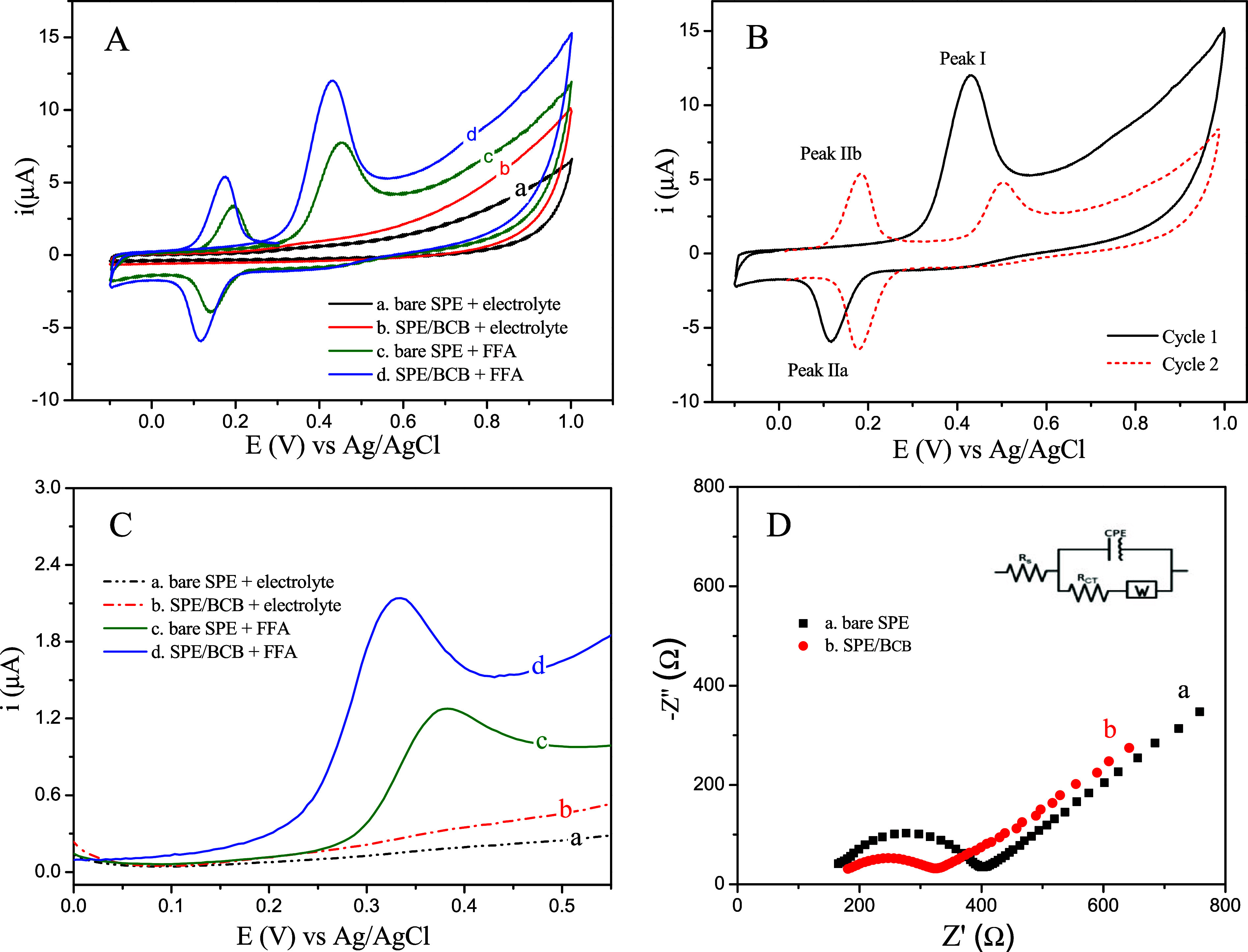
Cyclic voltammograms of 25 μmol L^–1^ FFA in 0.1 mol L^–1^ PBS (pH 7): (A) comparative analysis, (a, b) electrolyte, (c) bare SPE, and (d) SPE/BCB; (B) CV analysis for two cycles. DP voltammograms: (C) comparative analysis, (a, b) electrolyte, (c) bare SPE, and (d) SPE/BCB in FFA 6.60 μmol L^–1^ in 0.1 mol L^–1^ PBS solution. (D) EIS analysis for (a) bare SPE and (b) SPE/BCB in [Fe­(CN)_6_]^3–^/^4–^ 5 mmol L^–1^ in 0.1 mol L^–1^ KCl.

The DPV analysis was performed with both the bare SPE sensor and the SPE/BCB sensor in the presence of 6.60 μmol L^–1^ FFA in PBS 0.1 mol L^–1^ at pH 7, within a potential range of 0.0–0.6 V. The anodic response for FFA was enhanced, showing a current of 1.3 μA (0.38 V vs Ag/AgCl reference electrode in KCl_sat_) for the SPE/BCB sensor, compared to 2.2 μA (0.32 V vs Ag/AgCl reference electrode in KCl_sat_) for the bare SPE sensor. This enhancement is corroborated by the CV analysis, which demonstrates the synergistic contribution of the BCB material to the anodic and electrocatalytic responses of the FFA on the proposed electrode surface, as shown in Figure 3C.

Electrochemical impedance spectroscopy (EIS) analysis using [Fe­(CN)_6_]^3‑/4–^ 5 mmol L^–1^ in 0.1 mol L^–1^ KCl revealed two distinct charge transfer resistances (Rct) of 237 Ω for bare SPE and 147 Ω for SPE/BCB, indicating variations in electron transfer efficiency at the electrode–solution interface (Figure 3D). The resistance of 147 Ω is associated with more efficient charge transfer, reflecting faster interaction between the redox species and the electrode surface, while the resistance of 237 Ω suggests a slower process and lower surface activity in the bare SPE.

The influence of pH (5, 6, 7, and 8) was studied using differential pulse voltammetry (DPV) in the potential range of 0.025–0.7 V on the oxidation process of 10 μmol L^–1^ FFA, with the voltammograms presented in Figure 4. The decrease in hydrogen ion concentration in the PBS shifts the anodic peak potential to lower potential values. The linear correlations between peak potential (*E*_p_) and pH (*R* ^2^ > 0.98) presented a slope of 62 mV pH^–1^ (inset – Figure 4). In the correlation between peak current (*i*_p_) and pH expressed in the voltammograms (curves a–d, Figure 4), it is observed that, within the analyzed pH range, the maximum peak current value and defined response signal occurred at pH 7. This value was adopted for subsequent analyses.

**Figure 4:**
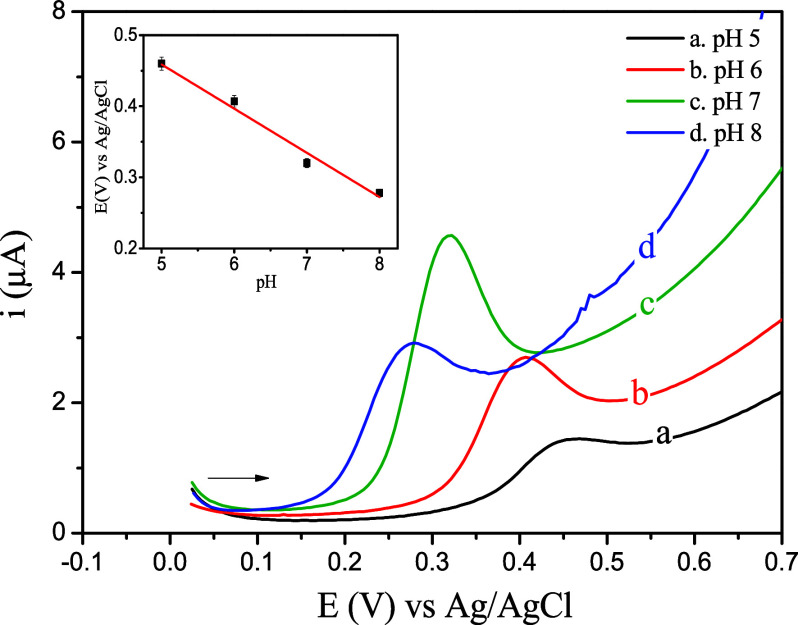
Effect of pH on the anodic response of the FFA 10 μmol L^–1^ in PBS 0.1 mol L^–1^ solution with the SPE/BCB sensor (inset: relation between pH vs E (V)).

According to the Nernst eq 1:

1 E(V)=−0.0592m/n+b

where the term 0.0592*m*/*n* is the angular coefficient of the linear correlation between peak potential (*E*_p_) and pH, *m*/*n* represents the ratio between the number of protons (*m*) and the number of electrons (*n*).^31,32^ Thus, it was concluded that the proton-to-electron ratio was 1:1 for the peak observed in the FFA oxidation process on the surface of the SPE/BCB sensor.

In addition, the influence of scan rates from 10 to 200 mV s^–1^ on the analytical response of the oxidation of 50 μmol L^–1^ of FFA was studied by the CV technique (Figure S2). The correlation between *i*_p_ and *v* (mV s^–1^) was linear (*R* ^2^ > 0.99), indicating that the process is adsorption-controlled (Figure S2B). In addition, the linear correlation between *E*_p_ and log v (V s^– 1^) (Figure S2C), with a slope value of 0.041 (*R* ^2^ > 0.98), confirms that the oxidation of FFA on the surface of SPE/BCB is adsorption-controlled, and the number of transferred electrons is one, according to Laviron’s eq 2:

2 $$E_{\mathrm{pa}}=E^{{}^{\circ}}+\frac{\mathrm{RT}}{\alpha nF}\ln\frac{{\mathrm{RTk}}^{{}^{\circ}}}{\alpha nF}+\frac{\mathrm{RT}}{\alpha nF}\mathrm{lnv}$$

The value of *n*, approximately equal to 1, was also calculated using eq 3, with α set to 0.5, which is typically applied to irreversible processes.^33^

3 $$E_{\mathrm{pa}}-E_{\mathrm{pa}/2}=\frac{47.7\mathrm{mV}}{\alpha n}$$

The data corroborate the electrooxidation of the FFA. The FFA contains a secondary aromatic amine group (−NH−). During the oxidation process, this amine group may undergo deprotonation (loss of H^+^), followed by the removal of an electron (e^–^). This sequential process leads to the formation of a nitrogen-centered radical (N•), as shown in Scheme 1, which is resonance-stabilized by the adjacent aromatic system.

### DPV Optimization Parameters

The optimization of the DPV technique parameters was carried out in the presence of 10.0 μmol of L^–1^ FFA in 0.1 mol of L^–1^ PBS (pH 7). The parameters evaluated were step potential, modulation amplitude, and modulation time. Initially, the step potential was varied at 1, 2, 3, 4, and 5 mV, with the best signal response observed at 3 mV. Next, modulation amplitudes of 10, 20, 50, 75, and 100 mV were tested, and the highest Δ*i*_p_ was obtained at 100 mV. Finally, modulation times of 10, 20, 30, 50, and 80 ms were examined, with 50 ms yielding the highest signal response for FFA oxidation. Therefore, the optimized parameters were 3 mV for step potential, 100 mV for modulation amplitude, and 50 ms for modulation time (Figure S3).

### Analytical Response

The detection performance of FFA electrooxidation using the SPE/BCB sensor was evaluated by the DPV technique within the potential range of −0.2–0.7 V, under the previously optimized parameters. The results obtained are listed in Figure 5. Figure 5 illustrates the linear relationship between *i*_p_ (μA) and [FFA], expressed by eq 4:

4 $$i_{\mathrm{pa}}\left(\mathrm{\mu A}\right)=2.30\mathrm{FFA}\left({\mathrm{\mu mol L}}^{-1}\right)+3.90;R^{2}>0.99$$

in the linear range from 0.05 to 13.32 μmol L^–1^. The limit of detection (LOD) was 1.30 nmol L^–1^, calculated using eq 5 and the limit of quantification (LOQ), using eq 6:

5 $$\mathrm{LOD}=3\times \frac{\sigma }{b}$$

6 $$\mathrm{LOQ}=10\times \frac{\sigma }{b}$$

where σ represents the average standard deviation of the blank (*n* = 10) and *b* is the slope of the calibration curve.

**Figure 5:**
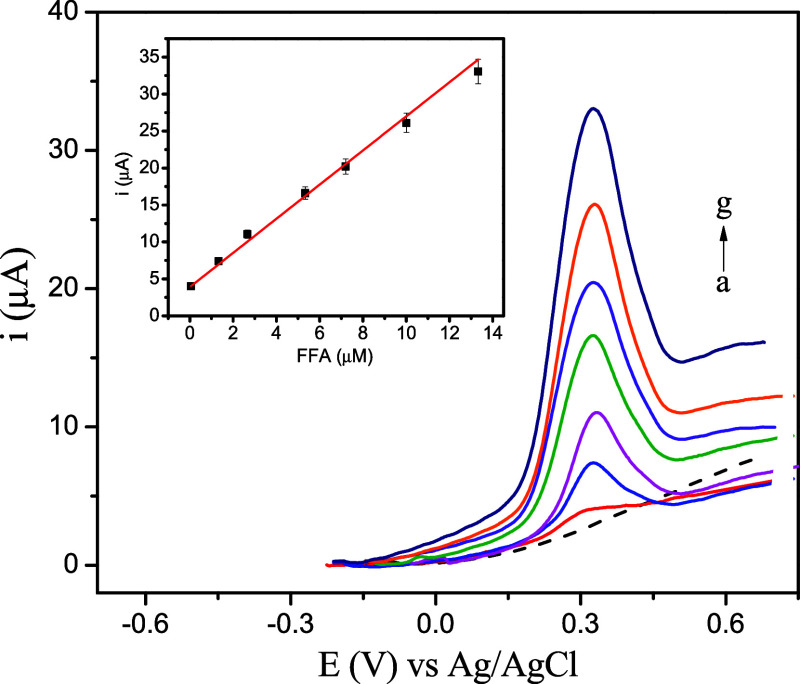
DP voltammograms at the concentrations: (a) electrolyte; (b) 0.05; (c) 1.33; (d) 2.65; (e) 5.30; (f) 7.20; (g) 10.01; and (h) 13.32 μmol L^–1^ (inset: correlation plot between FFA (μmol L^–1^) and *i*_p_ (μA).

According to the data presented in Table 1, the screen-printed electrode (SPE) modified with bamboo biochar (BCB) (proposed in the study) offers a cost-effective and sustainable alternative compared to conventional electrodes, such as glassy carbon electrodes (GCE) and pyrolytic graphite electrodes (PyGE), or those modified with expensive materials like multiwalled carbon nanotubes (MWCNTs) and ruthenium-doped TiO_2_ (Ru-TiO_2_). The SPE/BCB is not only affordable but also disposable, eliminating issues such as surface passivation or the need for constant maintenance of the modification, thereby reducing operational costs and improving ease of use. Its porous structure and high surface area provide excellent adsorption capacity, and as a carbonaceous material, it features various active sites that enhance the electrooxidation signal of FFA on the electrode surface, making it efficient in voltammetric techniques. The advantages of the SPE/BCB and the nanomolar LOD obtained demonstrate the efficiency of the sensor.

**Table 1:** Comparison of the SPE/BCB Sensor with Other Electrochemical Platforms for FFA Determination

Electrode	Technique	Medium/pH	Linear range (μmol L^–1^)	LOD (nmol L^–1^)	Refs
GCE/PNAANI^a^	CV^b^	BRB/2.5	2.5–9.0	5.7	^18^
PyGE/MWCNTs^c^	SWV^d^	PBS/4.2	0.1–0.8	2.2	^19^
PyGE/XAD-4+Ag-TiO_2_ ^e^	SWV	PBS/7.0	0.3–3.0	1.2	^20^
CPE/Ru-TiO_2_ ^f^	SWV	PBS/6.0	5.0–1000	1.7	^21^
CPE/MWCNTs+Ru-TiO_2_ ^g^	SWV	PBS/5.0	0.01–0.9	0.7	^22^
CPE/DTAC^h^	DPAdsV^i^	PBS/7.0	0.001–50	0.6	^23^
SPE/BCB^j^	DPV^k^	PBS/7.0	0.05–13.32	1.3	This work

a GCE/PNAANI – glassy carbon electrode/poly-*N*-acetylaniline.

b CV – cyclic voltammetry.

c PyGE/MWCNTs – pyrolytic graphite electrode/multiwalled carbon nanotube.

d SWV – square wave voltammetry.

e PyGE/XAD-4+Ag-TiO_2_ – pyrolytic graphite electrode/silver-doped titanium dioxide/Amberlite.

f CPE/Ru-TiO_2_ – carbon paste electrode/ruthenium-doped TiO_2_ nanoparticles.

g CPE/MWCNTs+Ru-TiO_2_ – carbon paste electrode/blend of ruthenium-doped TiO_2_ nanoparticles and multiwalled carbon nanotubes.

h CPE/DTAC – carbon paste electrode/dodecyltrimethylammonium chloride.

i DPAdsV – differential pulse adsorptive stripping voltammetry.

j SPE/BCB – screen-printed electrode/bamboo biochar.

k DPV—differential pulse voltammetry.

### Reproducibility and Stability

The reproducibility and stability further highlight the advantages of the SPE/BCB in the presence of FFA at 2.20 μmol L^–1^. Reproducibility was assessed by testing three electrodes, yielding a relative standard deviation (RSD) of 3.0% (Figure S4A), demonstrating consistent performance across multiple electrodes. Stability was evaluated over a 30-day period, with the RSD value recorded at 6.0% (Figure S4B), indicating that the electrode maintains reliable performance over time. All stability measurements were performed in triplicate to ensure accuracy and minimize experimental variability. These results confirm the robustness and reliability of the SPE/BCB sensor.

### Interferences Analysis

A selectivity study was conducted using the DPV technique to evaluate the potential interference of various substances in the determination of FFA at a concentration of 10.0 μmol L^– 1^ (reference). The analyses included dopamine (DA), ascorbic acid (AA), uric acid (UA), glyphosate (GLY), thiamethoxam (TMX), imidacloprid (IMI), flunixin meglumine (FXN), ivermectin (IVM), and gentamicin sulfate (GEN). These potential interferents were analyzed at a concentration ratio of 1:100 relative to FFA. The results, summarized in Figure 6, indicate that the presence of these substances did not produce significant interference, demonstrating the high selectivity of the proposed method.

**Figure 6:**
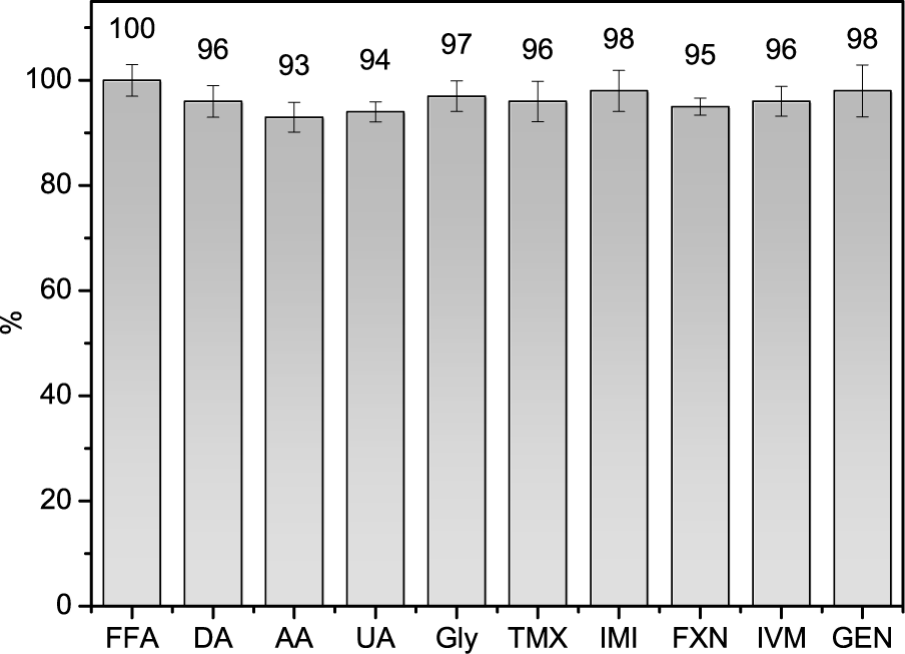
Selectivity studies for 10.0 μmol L^–1^ FFA in 0.1 mol L^–1^ PBS pH 7 (reference) in the absence and presence of possible interferent compounds in 100-fold higher concentrations.

### FFA Determination in Real Samples

The accuracy analyses were conducted using the DPV technique in tap water and river water samples via the standard addition method. The samples were prepared in a 0.1 mol L^– 1^ PBS at pH 7, with concentrations of 0.40, 5.68, and 8.52 μmol L^– 1^ for both studied matrices. The recovery values obtained ranged from 95 to 101% for tap water samples and from 90 to 96% for river water samples. The data demonstrate that the method provides consistent and accurate results, even in different sample matrices. These analyses were performed in triplicate, and the data are presented in Table 2. The recovery data were compared with those from the UV–vis method (Figure S5) at a confidence level of 95%. This comparison enabled the identification that there is no significant difference between the methods, demonstrating high reliability for the quantification of FFA in different matrices using the proposed method, with the calculated *t*-value being lower than the tabulated *t*-value.

**Table 2:** FFA Determination in Environmental Real Samples^a^

	DPV method			UV–vis	
Samples	Added (μmol L^–1^)	Found (μmol L^–1^)	Recovery (%)	Found (μmol L^–1^)	Recovery (%)
Tap water A	-	No detected	-	No detected	-
Spiked tap water B	0.40	0.38	95 ± 3	0.39	97 ± 3
Spiked tap water C	5.68	5.75	101 ± 2	5.56	98 ± 3
Spiked tap water D	8.52	8.30	97 ± 3	8.25	97 ± 2
River water E	-	No detected	-	No detected	-
Spiked river water F	0.40	0.36	90 ± 4	0.38	95 ± 5
Spiked river water G	5.68	5.40	95 ± 2	5.54	97 ± 2
Spiked river water H	8.52	8.20	96 ± 2	8.31	98 ± 2

a Student test: *t*_calc_ = −1.22 < *t*_crit_ = 2.57 at 95% confidence level.

## Conclusions

This study introduces an electrochemical platform for the detection of flufenamic acid (FFA), utilizing a bamboo biochar-modified screen-printed electrode (SPE/BCB). The proposed sensor demonstrated excellent analytical performance, exhibiting high sensitivity (2.30 μA/μmol L^– 1^) and an ultralow detection limit (1.3 nmol L^– 1^) across a broad linear range (0.05–13.32 μmol L^– 1^). Compared to conventional electrochemical sensors (such as glassy carbon electrodes, carbon paste electrodes, and modified pyrolytic graphite electrodes), the SPE/BCB sensor offers distinct advantages, including cost-effectiveness, ease of fabrication, and disposability. The incorporation of bamboo biochar not only enhances the electrochemical response but also aligns with environmentally sustainable practices. In addition to its high sensitivity, the sensor demonstrated remarkable selectivity, remaining unaffected by common organic interferents. This feature is especially relevant for environmental monitoring, as it enables accurate quantification of FFA in complex aqueous matrices, such as river and tap water. The combination of simplicity, affordability, and excellent analytical performance makes the proposed SPE/BCB sensor a promising alternative to existing electrochemical methods, highlighting its potential for applications in environmental analysis.

## Supplementary Material



## Data Availability

The data underlying this study are available in the published article and its Supporting Information.

## Notes

Cycles voltammograms in two different potential ranges; scan rate analysis; parameter optimization of the DPV technique; and DPV data for reproducibility and storage stability (PDF)
