# Supplementary material for: A Novel Disposable Bamboo Biochar-Based Electrochemical Sensor for Detecting the Nonsteroidal Anti-Inflammatory Drug Flufenamic Acid in Environmental Samples
Source: ACS Omega. 2025 Aug 5;10(32):36724–32. doi: 10.1021/acsomega.5c06160 (PMC12368646; doi:10.1021/acsomega.5c06160)
Supplement: Supplementary file 1 [file ao5c06160_si_001.pdf]

Supplementary material

A novel disposable bamboo biochar-based electrochemical sensor for detecting the non-steroidal anti-inflammatory drug flufenamic acid in environmental samples

Francisco Walison Lima Silva[1]; Luís Eduardo da Conceição Teixeira[1]; Cassiano Augusto Rolim Bernardino[2]; Claudio Fernando Mahler[2]; Renata Coura Borges[3], Ricardo Erthal Santelli[1,4] and Fernando Henrique Cincotto[1,4*]

[1] Departamento de Química Analítica, Instituto de Química, Universidade Federal do Rio de Janeiro, Rio de Janeiro, 21941-909, Brazil.
[2] Departamento de Engenharia Civil, COPPE, Universidade Federal do Rio de Janeiro, Rio de Janeiro, 21941-914, Brazil.
[3] Departamento de Solos, Instituto de Agronomia, Universidade Federal Rural do Rio de Janeiro, Rio de Janeiro, 23897-000, Brazil.
[4] National Institute of Science & Technology of Bioanalytics (INCTBio), Campinas-SP, 13083-970, Brazil.

*Corresponding author
E-mail address: fernandocincotto@iq.ufrj.br; fernandocincotto@gmail.com

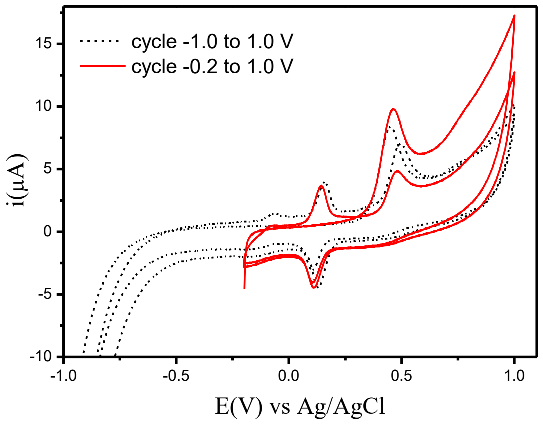

Figure S1 – CV comparative from cycles in two different potential range.

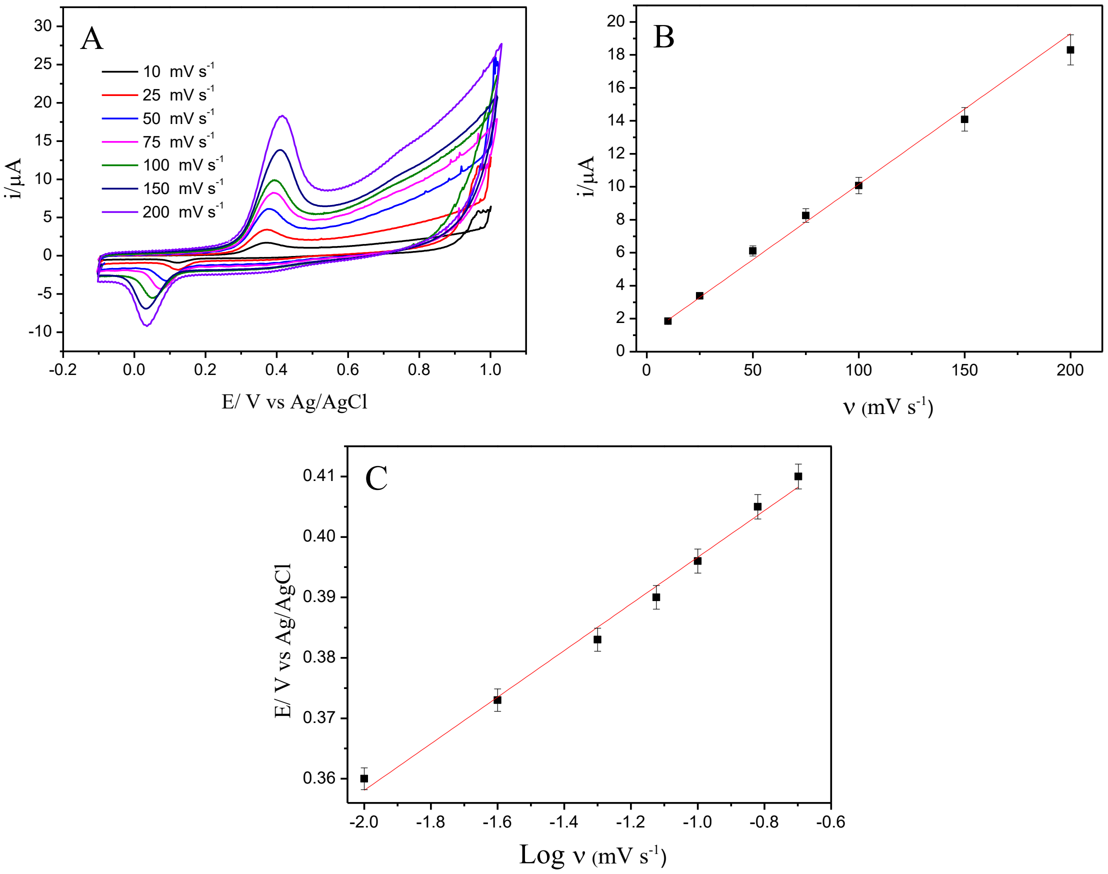

Figure S2 - (A) Voltammograms of the different scan rates in the presence of FFA. (B) correlation linear between i_{pa} and v. (C) correlation linear between E_p and Log v.

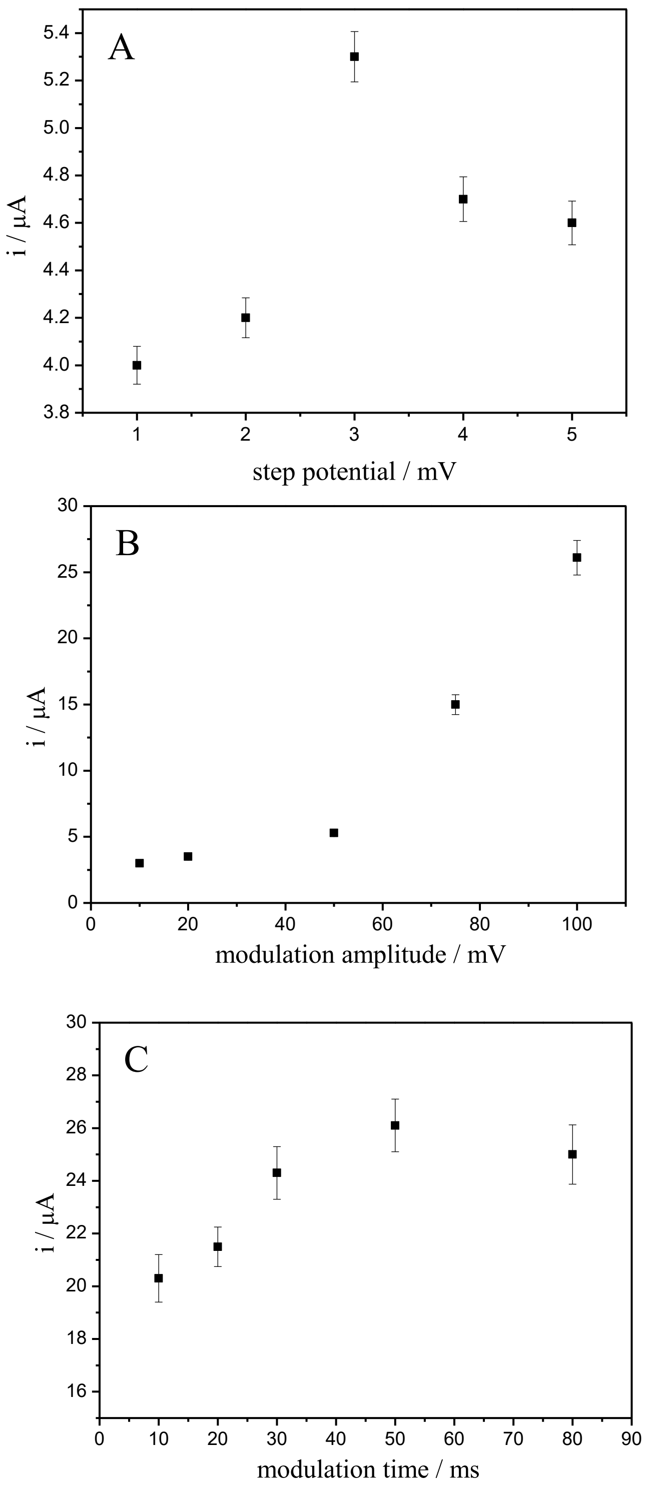

Figure S3 - Graphs of optimization de parameter: (A) step potential, (B) modulation amplitude and (C) modulation time (FFA 10.0 µmol L^{-1}).

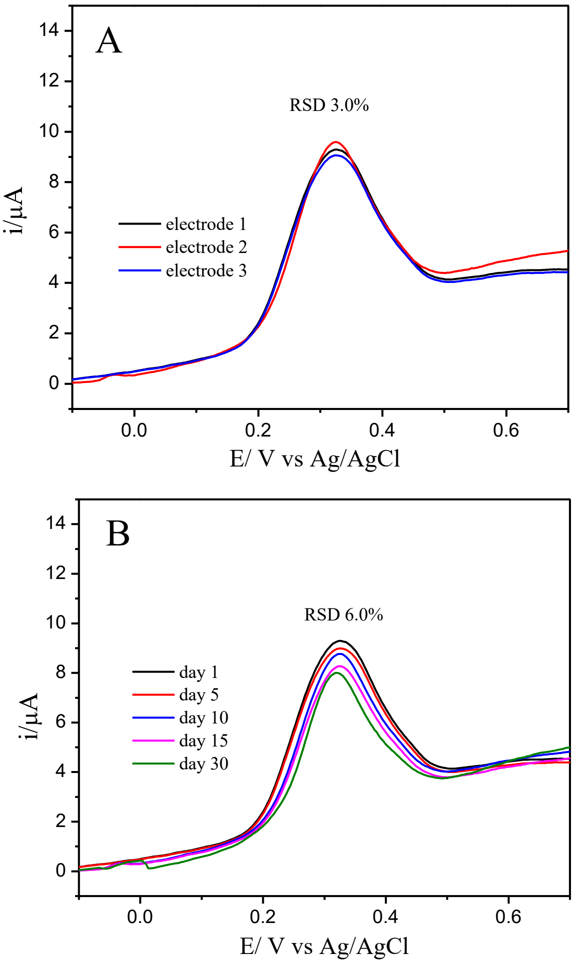

Figure S4 - DPV results for the (A) reproducibility, (B) storage stability for 2.20 μmol L^{-1} in PBS 0.1 mol L^{-1} pH 7.

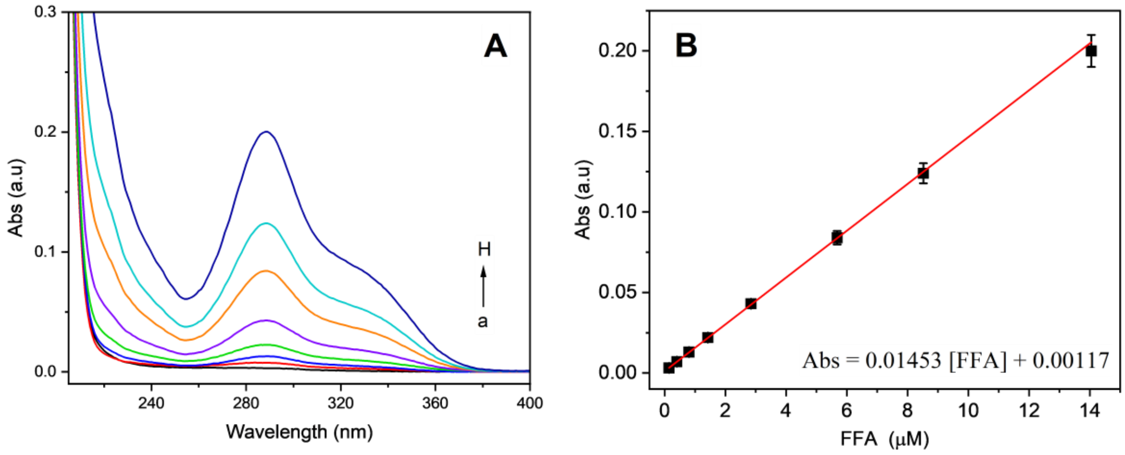

Figure S5 – (A) UV-vis spectra at concentrations: (a) 1.41; (b) 0.4; (c) 0.8; (d) 1.42; (e) 2.84; (f) 5.68; (g) 8.52 and (h)14.05 μmol L^{-1}. (B) calibration curve**.**
